# Can exercise shape your brain? A review of aerobic exercise effects on cognitive function and neuro-physiological underpinning mechanisms

**DOI:** 10.3934/Neuroscience.2022009

**Published:** 2022-04-02

**Authors:** Blai Ferrer-Uris, Maria Angeles Ramos, Albert Busquets, Rosa Angulo-Barroso

**Affiliations:** 1 Institut Nacional d'Educació Física de Catalunya (INEFC), Universitat de Barcelona (UB), Barcelona, Spain; 2 Department of Kinesiology, California State University, Northridge, CA, United States

**Keywords:** physical activity, cognition, learning, animals, young adults, children, brain, neuro-physiological mechanisms, school exercise-intervention, workplace exercise-intervention

## Abstract

It is widely accepted that physical exercise can be used as a tool for the prevention and treatment of various diseases or disorders. In addition, in the recent years, exercise has also been successfully used to enhance people's cognition. There is a large amount of research that has supported the benefits of physical exercise on human cognition, both in children and adults. Among these studies, some have focused on the acute or transitory effects of exercise on cognition, while others have focused on the effects of regular physical exercise. However, the relation between exercise and cognition is complex and we still have limited knowledge about the moderators and mechanisms underlying this relation. Most of human studies have focused on the behavioral aspects of exercise-effects on cognition, while animal studies have deepened in its possible neuro-physiological mechanisms. Even so, thanks to advances in neuroimaging techniques, there is a growing body of evidence that provides valuable information regarding these mechanisms in the human population. This review aims to analyze the effects of regular and acute aerobic exercise on cognition. The exercise-cognition relationship will be reviewed both from the behavioral perspective and from the neurophysiological mechanisms. The effects of exercise on animals, adult humans, and infant humans will be analyzed separately. Finally, physical exercise intervention programs aiming to increase cognitive performance in scholar and workplace environments will be reviewed.

## 1. Introduction

New scientific evidence is added daily to justify the prescription or recommendation of regular physical activity practice. Physical exercise has been shown to be a primary tool in the prevention and treatment of diseases or disorders of very diverse origin such as obesity [1], colon or breast cancer [2], Parkinson disease [3],[4], developmental coordination disorder [5],[6], attention deficit hyperactivity disorder (ADHD) [7],[8], learning impairment [9], or in a multitude of psychological problems, such as depression and anxiety [10].

Beyond its function as an instrument for the prevention or treatment of disorders and pathologies, physical activity, and especially aerobic exercise, has been attributed the property of positively influencing cognitive performance [11]–[14]. Cognition or cognitive function can be defined as a set of mental abilities that can be grouped into areas or dimensions, among which we highlight executive function (including inhibition, cognitive flexibility, planning and execution, and updating short-term memory), attention and memory, as they are closely related to learning capacity [15]–[18]. In this way, the practice of physical exercise could be a tool for the stimulation of cognitive function and learning capacity [16],[19],[20], either from regular exercise (long-term exercise or training) or from participation in a single session of physical exercise (acute exercise).

To understand how aerobic exercise affects cognitive function, researchers have not only studies changes in behavior but have also focused on the biological mechanisms that underpin these changes. Thanks to advances in neuroimaging techniques, there is a growing volume of scientific evidence that allows determining the anatomical and functional changes that occur in different brain areas and the magnitude of the benefits that exercise can induce related to cognitive function [3],[21],[22]. Greater difficulties exist when trying to investigate the physiological effects of exercise on the human brain due to the high degree of invasiveness that data collection techniques would entail. For this reason, the direct observation of exercise effects on brain's physiology is usually approached with animal studies [23], while indirect evaluation in humans is usually approached through analysis of blood biomarkers [24].

Knowledge of the benefits of physical exercise and the neuro-physiological mechanisms involved could provide a greater scientific basis for the development of intervention plans based on increasing regular aerobic physical exercise to enhance cognitive performance and even academic and professional performance. In addition, the findings derived from acute exercise interventions may be especially important for their specific use in populations that are in their formative stages or in situations where the improvement of cognitive function would especially benefit their learning capacity, for example, in elementary schools, middle or high schools, or even university level.

Thus, below, some of the latest advances in the study of the effects of aerobic physical exercise on cognitive function (especially the effects on these dimensions of cognitive function most associated with learning capacity) and learning capacity will be reviewed in child and youth (6–17 years) and young adult (18–35 years) ages. The results obtained from studies with long-term interventions (training) will be differentiated from those obtained from participation in a single session of physical exercise (acute exercise). Likewise, knowing the existing difficulties in studying the physiological effects of exercise on the human brain, we will also rely on studies carried out in animals to expose the biological factors involved in the exercise-cognitive capacity association. Finally, after analyzing the state of the question, we will present some successful intervention studies aimed at improving cognitive function and learning capacity in young adult populations and children in specific contexts (i.e., current work and academic, respectively).

## 2. Long-term exercise (training) effects on cognitive function

### 2.1. Effects on animals

Studies of the effect of regular exercise on cognitive function carried out in animals have made it possible to observe directly (from their dissection and extraction of blood and brain tissues) and indirectly (via neuroimaging techniques) the physiological alterations that happened to the brain. Through these observations, it has been proven that access to means for the practice of exercise, such as a running wheel, entails structural changes and an increase in the functionality of specific brain regions, such as the hippocampus, a region where memory formation originates [25]. It has been observed that these improvements in brain structures could be mainly due to three factors: (1) increased secretion of neurotrophic substances that promote neurogenesis or increase in the number of neurons [25]–[30], (2) angiogenesis or increase in vascularization [31],[32], and (3) increased synaptic plasticity (synaptogenesis) or the organism's ability to create new connections between neurons [32],[33]. Thus, based on these factors, it seems that aerobic exercise has the ability to structurally affect the brain and therefore improve certain dimensions of cognitive function, such as executive function and memory, and consequently, the ability to learn and improve motor execution in animals [25],[32]–[38]. In addition, the preventive and therapeutic effect of aerobic training in mitigating memory impairment in situations such as chronic stress has been examined and confirmed [39].

A clear example of these cognitive improvement phenomena is the report by Vaynman et al. [25] where the mice that had access to an exercise wheel showed an increase in the production of neurotrophic substances and in the formation of new neuronal connections at the hippocampus level. This fact was related to their faster learning and better memorization of the maze structure. Likewise, in a recent work by Martínez-Drudis et al. [23], where rats' brains were injured, they began a 25-day program of voluntary aerobic physical exercise eleven days after the injury. The exercise served as a restorer of memory functions damaged by the induced brain injury. In addition, the concentration of the neurotrophic substance BDNF (brain-derived neurotrophic factor) increased in the hippocampus and correlated positively with the temporal order recognition memory capacity, but not with the object location recognition memory, evaluated with objects such as Lego pieces or beverage cans which varied in shape, color and size.

Neuroimaging studies appear to corroborate these long-term effects of aerobic exercise. Thus, rats that voluntarily ran on an exercise wheel, demonstrated an increase in the gray matter of the hippocampus [40] and an increase in the cerebral blood volume in structures involved in the formation of new neurons (e.g., the dentate gyrus) [21].

On the other hand, other studies have observed that improvements in cognitive function and neuro-physiological changes were subject to parameters of the training load such as frequency, duration, intensity or type of exercise [36],[41],[42]. For example, and in relation to intensity, some studies have observed how low intensity exercise (light walking) reinforced some cognitive areas such as spatial learning and memory [43], while some other neurophysiological changes such as neurogenesis, is more reinforced as the intensity of the exercise increases [35].

### 2.2. Effects in human adults

In studies carried out with humans, aerobic physical exercise has been shown as a potential tool to promote the improvement of different areas of human cognition and in populations of different ages [14],[17],[19],[44]. Using neuroimaging techniques and indirect measures to assess brain function, it has been observed in adult humans how aerobic physical exercise has a direct effect on the biological mechanisms underlying cognitive improvements, very similar to those seen in animal studies. Changes in brain structure and function in adult humans are also due to the three previously mentioned mechanisms: neurogenesis, angiogenesis and synaptogenesis [21],[45]. It is thought that these processes are mainly regulated by neurotrophic substances (e.g., BDNF) [46],[47] and growth factors (e.g., insulin-like growth factor 1, IGF-1) [48],[49]. It seems that the concentration of these substances is altered by long-term aerobic exercise. In the case of BDNF, its level in serum and plasma is increased after aerobic training [46]. Feter et al. [47] conducted a systematic review, meta-analysis and meta-regression on the different parameters of the training load that modulate the neurotrophic factors' blood concentration levels. Based on their conclusion, and to increase BDNF concentration levels, they proposed performing aerobic exercise with a minimum intensity of 65% of VO2max (i.e., moderate-high intensity), with a frequency of 2–3 times per week, and a duration of at least 40 minutes of continuous training per session.

In addition, evidence suggests that a greater amount of exercise and better cardiovascular performance is related to the volume of various brain structures, such as the hippocampus [50]–[52] and the basal ganglia [53], both involved in the formation of memory and learning. This relationship between cardiovascular performance and brain volume was reaffirmed in a randomized clinical trial by Erickson et al. [51]. In this study, a 12-month aerobic physical exercise program intervention resulted in a 2% increase in the volume of the hippocampus, which was related to an increase in the secretion of neurotrophic substances (measuring their concentration in the blood). The authors indicated that these changes could positively affect the improvement in the participants' memory and learning. Such cognitive function benefits appear to be modulated, like in animal findings, by the duration and intensity of the training program and the frequency of the sessions [46]. Griffin et al. [19] showed how a program of three weekly sessions of 30 to 60 minutes on a cycle ergometer at an intensity close to 60% of VO2max, managed to increase aerobic performance, the secretion of neurotrophic substances, and the learning capacity when the program was executed during 5 weeks. Conversely, the aforementioned benefits were not achieved if the exercise program was disrupted after the third week. Thus, it seems that the optimal effects on brain structures are obtained when training lasts longer than three weeks and the intensity of exercise is moderate to high [11],[19],[54],[55]. Furthermore, it appears that a frequency of three training sessions per week is already effective in inducing neuronal changes [46].

Aerobic training interventions of the previously mentioned characteristics have demonstrated a positive effect on specific cognitive dimensions [11],[17],[56], especially in: (1) executive function, (2) attention span and information processing speed, and (3) memory. In the same way, learning capacity, which is closely related to the mentioned cognitive dimensions, is also positively affected by training since the increment of neuro substance like BDNF due to the exercise practice, favors an stimulating environment for neural plasticity [48],[57]. For instance, focusing on motor learning, aerobic training increases the excitability of the cortical response of the muscles involved during the learning of the motor skill. This implies that the combination of aerobic training with the practice of motor skills improves motor learning to a greater extent than the performance of the isolated motor skill practice [58],[59].

Nevertheless, and from a statistical point of view, the magnitude of the exercise training effects on cognitive functions found in systematic reviews examining this subject has been discrete (see reviews by: Roig, Nordbrandt, Geertsen, et al. [55]; Smith et al. [17]). Therefore, if exercise is capable of inducing positive adaptations at the brain level, why are the changes produced on cognitive function in adults relatively small? It seems that the answer to this question lies in the state of maturation of the nervous system and initial cognitive performance. Young adults are shown to be in the vital moment of maximum cognitive performance [60], thus having a reduced margin of improvement due to an exercise intervention [61]. In contrast, in children and elderly populations, cognitive performance appears to be conditioned by a maturing or declining nervous system [61]. Thus, can an aerobic training intervention increase cognitive performance in child populations to a greater extent than in adult populations?

### 2.3. Effects on humans of child and youth age

Through several studies with school-age participants, it has been observed that greater volume of physical activity practice and, in turn, better state of physical condition are positively related to greater cognitive performance and better academic performance [62]–[68]. Furthermore, benefits of physical activity on cognitive dimensions such as executive function, working memory, and attention have been consistently observed [11],[12],[16]. It seems that, as in the case of adults and animal studies, this relationship is not fortuitous, but is due to an improvement in brain functionality due to physical exercise practice. Children and adolescents' maturing brains are in one of the most sensitive stages to environmental stimulations [4], and physical exercise interventions are a good example of such stimuli. As a consequence of better physical condition or greater physical activity, studies of the brain through neuroimaging techniques have shown changes in brain regions involved in some dimensions of cognitive function (see Meijer et al. [69], for a systematic review and meta-analysis), such as greater integrity of the white matter and greater volume of the hippocampus [70],[71] or greater volume of the basal ganglia [11],[72]. In turn, the maturation and/or transitory improvement of the prefrontal cortex performance could be also facilitated [15]. Additionally, it has been demonstrated that a better state of physical condition is also related to a better distribution of attentional resources [73],[74] and a decrease in the latency of impulses' transmission through the brain [75].

Beyond its association with fitness level, cognitive and academic performance have been shown to benefit from children's participation in an aerobic training program. In a review, Tomporowski et al. [20] explained how, despite the small number of existing randomized clinical trials, interventions through physical activity, of an aerobic nature and with moderate to high intensity, contributed positively to the improvement of cognitive function, especially executive function, and children's academic performance. This increase in academic performance due to physical exercise has been demonstrated independently of the cognitive performance level prior to the intervention. However, the effect of exercise seems not to be independent of the level of aerobic performance and degree of participation in physical activity before the intervention. More specifically, it has been observed that BMI is a moderator of the effect of exercise on children's executive function, being those individuals with higher BMI those that obtained larger benefits from exercise interventions [76]. This phenomenon could be explained by a worse initial cognitive performance in overweight children [76]–[78], as well as a greater response to exercise due to their low fitness level.

Thus, it seems that aerobic training of moderate to high intensity has the ability to positively affect the young brain, especially the prefrontal cortex and the executive functions, both of which are in a maturation state well below that of adults [15],[20]. Nevertheless, more studies are needed to improve our understanding of the relationship between physical exercise interventions and cognitive performance in children.

## 3. Acute effects of exercise on cognitive function

### 3.1. Effects on animals

Although to date the majority of studies looking at the effects of exercise on cognitive function in animals have used long-term exercise interventions, there is a growing number of studies focused on the use of acute exercise as an intervention. Several publications have shown how acute exercise can be a precursor of (1) neurogenesis, increasing the levels of neuron growth regulating proteins or factors such as BDNF [77]; (2) angiogenesis, increasing growth factors that are fundamental in the improvement of the cerebral vascular system [79]; (3) synaptogenesis, for example, increasing the synaptic activity of the hippocampus [77]. For example, acute exercise sessions seem to produce positive effects on the regulation of the expression of neurochemicals (e.g., lactate or cortisol), neurotrophic substances (e.g., BDNF) and growth factors (e.g., IGF-1) [80], producing improvements in brain structures such as the hippocampus by increasing its neuroplasticity [77]. Furthermore, it has been suggested that acute doses of exercise may have a lower stress response than chronic exercise (i.e., without restricting access to a treadmill), since a high stress response would be detrimental to cell proliferation in the hippocampus [81].

Likewise, these neuro-physiological improvements produced by acute exercise seem to favor the enhancement of cognitive dimensions such as memory, thus facilitating learning [77],[82]. For instance, Fernandes, Soares, do Amaral Baliego, & Arida [83], examined the effects of a single exercise session on consolidation in memory of fear conditioning. They showed for the first time that a single exercise session applied just after conditioning increased contextual memory in rats. On the other hand, studies such as da Silva de Vargas, Neves, Roehrs, Izquierdo, & Mello-Carpes [84] and Rossi Daré, Garcia, Neves, & Mello-Carpes [85], also evaluated the effects of acute exercise on memory but, in these cases, they focused on object recognition memory. They found that, acting through the hippocampal noradrenergic mechanisms, a single session of aerobic exercise presented immediately after performing the encoding phase of an object recognition memory task, improved learning and memory persistence after 24 hours, 7 and 14 days. Therefore, these authors proposed acute exercise as a non-pharmacological intervention, without side effects, that may help in the consolidation and persistence of memory.

Finally, exercise parameters, such as intensity, duration or type of exercise, should be considered as they may produce different cognitive effects depending in their variation. For instance, in the case of acute exercise and reviewing exercise intensity, it has been shown that high levels are even more beneficial for neurogenesis and cell survival in the dentate gyrus than moderate intensities [86].

### 3.2. Effects on adults

Beyond the benefits of a training program in cognitive performance, it has been shown that a single session of aerobic physical exercise can positively affect human cognition [19],[87]. There is evidence that acute aerobic exercise can promote an optimal environment for the development of neuroplasticity [88] and positively and selectively affect the connectivity and functionality of various brain structures such as the hippocampus, motor cortex or prefrontal cortex [89]–[92]. In addition, it has been observed that acute aerobic exercise can positively affect several cognitive dimensions [93] and learning processes [94],[95]. For instance, a single exercise session has been shown to produce improvements in executive function and attention [96]–[99], or memory formation processes [55],[100]–[104].

It seems that the mechanisms by which acute exercise can produce these cognitive and learning process improvements are similar to those induced by aerobic exercise training, mainly an increase in the secretion of neurotrophic substances and neurotransmitters and an increase in blood flow to the brain [55],[67],[105]. Results from studies suggest that peripheral BDNF blood concentration is increased after a single session of exercise [105],[106]. Furthermore, exercise would produce metabolites like lactate and the ketone body β-hydroxybutyrate (DBHB), which have the ability to cross the blood-brain barrier, act on the nuclear protein SIRT-I and inhibit class I HDACs, which activates the expression of cerebral BDNF [105],[106]. Elevated cerebral BDNF concentrations may result in increased neuronal growth, survival, and synaptogenesis, leading to the cognitive and affective benefits observed after exercise. On the other hand, increased cerebral blood flow is also thought to be responsible of the exercise-enhancing effect on cognition due to its relation to the oxygen availability to the brain [67]. However, recent evidences have shown that altered cerebral blood flow via hypercapnic respiratory gas during acute exercise did not alter cognitive performance [107] or moderate exercise-induced cognitive benefits [108]. These authors propose that exercise benefits on cognition might be regulated by multiple factors but not by exercise-induced alterations in blood flow [108]. Even so, the benefits obtained through this type of intervention seem to depend on certain moderators such as the characteristics of the exercise (intensity, duration, and type), the person's level of cardiovascular physical condition, the cognitive task to develop or learn, the time elapsed between the exercise session and the cognitive task, and the temporal order in which they occur [109]–[112].

The characteristics of exercise could be one of the most relevant moderators, since they seem to have a very direct relationship with the secretion of neurotrophic substances [46],[54],[55],[104],[110],[111],[113] and the modulation of certain brain areas [89],[114],[115]. In relation to the exercise intensity, it seems that the greatest benefits would be obtained from interventions of moderate to high intensity, while exercise of too low intensity has been shown to have a very limited effect at the cognitive function level [54]. Thus, a moderate intensity exercise would produce the greatest benefits in cognitive dimensions such as executive function [54],[115], while other dimensions like memory or attention could benefit more from a higher intensity exercise [55],[104],[116]–[119]. Regarding the exercise duration, it should be taken into account that an excessively long exercise can generate excess fatigue and at the same time cause an excessive degree of dehydration, thus interfering with cognitive performance and also causing a negative effect of exercise on cognition [120]. Similarly, too short exercise bouts may not represent a sufficient stimulus to enhance cognition [11],[121]. Recent reviews have observed that best cognitive enhancements were obtained between 11 and 21 min when exercising at moderate-to-vigorous intensity [11], and between 10 to 30 min when performing aerobic high intensity interval training type of exercises [121]. However, one has to consider that there are few studies that have used exercise durations outside of these ranges, especially on the lower end, and that little is known regarding the moderating mechanisms related to exercise duration. Finally, it seems that benefits from acute exercise were more frequent when exercise type was cycling compared to other forms of exercise (mainly running) [121],[122]. Although some studies, performed with children and adolescents, have argued that cognitive engaging exercises could have a greater impact on cognition [15],[123],[124], adult studies have found contradictory results [125]–[127]. Furthermore, it is difficult to argue that cycling is more cognitively engaging than running, although one could think that cycling could be a novel movement pattern for some people while running might be more familiar for everyone.

In relation to the moderating effect of fitness level, several studies have analyzed the effect of cardiovascular fitness level on brain function and cognitive performance [115],[128],[129]. Some of these studies have observed the modulatory effect of the fitness level in the activation of specific brain regions, such as the anterior cingulate cortex [130],[131] and prefrontal and parietal areas [130] and in the cognitive performance, such as executive function [132] or spatial learning tasks [131]. In addition, better aerobic capacity has also been positively associated with potential structural effects in the brain such as the viscoelasticity of the hippocampus, which positively correlates with memory performance [21],[133]. These factors, as has been reviewed above, predispose higher fit individuals to a better cognitive functioning. These pieces of evidence have led to belief that fitness level could have a positive modulating effect on the relationship between acute exercise and cognitive performance. However, one could think that those individuals with lower fitness and thus lower baseline cognitive performance could have a greater opportunity to benefit from acute exercise interventions. Conversely, evidence shows that those individuals with higher fitness level present greater effects on cognitive function when exposed to acute exercise than those with lower fitness level [55],[121]. Although it's an underdeveloped topic it could be that higher fit individuals present a better tolerance to aerobic exercise effort, thus experiencing lesser fatigue-related adversities immediately after the exercise. According to this thought, a meta-analysis by Chang et al 2012 observed that higher fit individuals presented better cognitive stimulation compared to lower fit when cognitive function was assessed either during or immediately after a bout of exercise. In fact, in a similar example, it has been observed how the intensity of exercise does not affect the stimulation of attention in athletes in the same way compared to non-athletes [134]. More specifically, athletes continued increasing attention with higher exercise intensities while non-athletes' attention showed an inverted-U curve. That is, non-athletes attention increased as exercise intensity did, finding the greatest level in attention with moderate exercise intensities, but decreased when the exercise intensity became higher. Additionally, fitness level could also prime neurobiological mechanisms like baseline production of BDNF which could also explain the moderator effect of fitness level on the exercise-cognition relationship [93],[135]. In this way, the regular practice of physical exercise could not only have a direct effect on cognition, but could also enhance the effects that acute exercise has on cognitive performance [136]. Still, further research is needed to be able to replicate these results and generalize them to other cognitive dimensions.

Regardless of the characteristics of the exercise and the level of physical fitness, the type of cognitive task, the temporal proximity and the temporal order of the cognitive task and exercise can also act as moderators of the acute exercise-cognition relationship. It has been observed that for the effects of exercise on cognition to be relevant, the cognitive task must be difficult enough to be challenging, complex to attract attention, and specific to the needs of the population (e.g., a task that requires motor circuits in the case of Parkinson's patients) [4],[137]. Considering the time elapsed between the exercise session and the cognitive task, there is evidence that the effects of exercise are dependent on the time passed between these two events [138]. Excessively long times between the task and the exercise can result in a null effect, although there is controversy in the literature about the size of the effective time window and the optimal timing between the two [139],[140]. Van Dongen et al. [141] found that best exercise-induced benefits on the recall of episodic memory were seen when the exercise bout was performed 4 hours after the task's encoding phase, compared to immediate exercise or non-exercising. Conversely, other studies have shown better exercise-derived benefits when memory tasks with motor components were temporally closer to the exercise bout, even presenting a time gradient effect of the exercise benefits on memory recall [87],[140]. Furthermore, a recent review has shown that benefits from high intensity aerobic exercise interventions on executive function only occurred within a window of 30 min from exercise performance [121]. Therefore, it seems that up-to-date, the best results could be expected when exercise and the cognitive task are presented in close temporal relation. However, further research is needed to understand the mechanisms underlying the optimal time-widow where exercise could benefit cognition and their interaction with other moderators like exercise intensity or cognitive task type. Lastly, studies exploring the effect of the temporal order between the exercise session and the cognitive task observed that exercising before an episodic memory test produced greater effects on short- and long-term memory than performing the exercise during or after the task [142]–[145].

### 3.3. Effects on humans of child and youth age

In the same way as in adults, in the case of children, it has been observed that acute aerobic exercise can induce improvements in cognitive capacity [20],[67],[146]. It seems that the cognitive dimension that is most susceptible to the effect of acute exercise is executive function, where those individuals who experience changes in development, such as young children and pre-adolescents, show the greatest sensitivity and a positive response to exercise [15],[147],[148]. Likewise, there is also evidence that an acute exercise intervention can positively influence long-term memory formation processes [149],[150], and according to neurophysiological correlates, can contribute to an optimal allocation of attentional resources [139].

Regarding the moderators that seem to regulate the effects of exercise, it has been observed that the best results are obtained from an exercise intervention of moderate intensity and a duration of between 10 and 50 min [146],[151]. However, there are reports that intense exercise bouts of only 5 min can enhance memory consolidation of a motor learning task [152]. On the other hand, it seems important to pay attention to the level of physical condition, since those children with higher levels of physical condition obtain greater cognitive benefits from the performance of acute exercise, than their lower fit counterparts [143],[151]. Thus, Oberste et al. [144] examined the role of cardiovascular fitness level in cognitive function, specifically in interference control performance, finding that individuals with a high level of physical condition benefited more from exercise than those individuals with a medium or low level.

In addition, another important moderator of the effect of exercise on cognition could be the cognitive activation that exercise itself entails. It has been observed that, from the use of exercise tasks that include a greater cognitive implication (such as team games, tasks involving decision making, ...), the effect that exercise produces in cognitive capacity is greater compared to the effect produced by an exercise task that aims only at the physiological activation of the organism [15],[124],[153]. However, it should be borne in mind that in this type of exercise, the level of intensity and participation of the children can be more difficult to control. For example, Jäger et al. [154] demonstrated that in an exercise intervention avoiding laboratory tasks and using tasks more similar to children's real play through a physical education class session, only those participants who had higher fitness level showed an improvement in executive functions. These results could be explained by the difficulty in individualizing the intensity of the exercise during the game, so that for some participants the exercise could have been too high or too low, causing the non-manifestation of the benefits [54]. Another possible explanation could be found in the differences found in the effect of exercise on executive functions according to the child's level of cognitive development [15]. Depending on the child's development and the type of cognitive involvement that is included in the exercise, we could find diverse and even adverse results, especially if the exercise turns out to be too cognitively demanding [151]. According to Best [15], in pre-adult ages, executive function may present a temporary period of greater sensitivity to acute exercise, and the different components of executive functions (i.e., inhibition, updating short-term memory, cognitive flexibility) may also have different sensitivity to exercise. Thus, Best [15], based on the findings from other studies [155]–[157], proposes that young children would be more sensitive to exercises where the executive function involved inhibition than exercises intended to affect the cognitive flexibility component of executive function, while the opposite would happen in adolescents. Still, to date, this is a controversial topic that needs further exploration.

Finally, we highlight that research is not only focusing on the use of this type of intervention in typically developing children, but also in children with special educational needs caused by various disorders [158]–[161]. An example of this research is the study of the effects of exercise on the cognitive capacity of children diagnosed with ADHD where improvements in the behavioral and cognitive symptoms of the disorder have been observed [7],[109],[162],[163]. Physical activity, whether acute or chronic, has even been suggested as a treatment to improve brain development in this population [164], given the possible regulation of neurotransmitters involved in this disorder, such as dopamine and norepinephrine [162],[165]. Seeing that an acute aerobic exercise intervention shows positive effects, both in children with disorders and in children with typical development, it seems that exercise could be a valid tool for cognitive stimulation in both populations.

## 4. Exercise interventions in the workplace and the classroom

Given the cognitive benefits shown by aerobic physical exercise from both acute interventions and training interventions, we support their use as strategies to enhance cognitive performance. However, interventions with physical exercise seem sometimes very difficult to implement due to the current context where individuals have tight schedules organized around what is considered their main function in society: (1) production at work for adults, and (2) formal learning in school for children and adolescents. In addition, current work and academic contexts bring individuals to maintain sedentary life styles that entail obvious adverse consequences for their health and well-being [166] and, likely, their cognitive abilities due to lack of exercise-based stimuli that facilitate their development and/or their maintenance [167].

Several studies have analyzed the possible effects of exercise on cognition and on school or work performance. Training interventions in the workplace context have proposed the introduction of exercise during work breaks. Initially, the proposal to carry out physical exercise during work breaks aimed to improve workers' health, job satisfaction and increase productivity [168],[169], but its positive effects on cognition have also been demonstrated [170]. On the other hand, in the academic context, exercise intervention proposals have been usually based on increasing the weekly volume of physical education throughout the pre-university academic stage. In a review, Trudeau & Shephard [68] observed that with a 40-minute increase in physical education per week, students tented to improve their academic performance. This academic improvement was also observed in the studies where the volume of weekly physical education hours increased, despite reducing the number of hours devoted to other subjects. On the contrary, the overall academic performance of students worsened when the volume of physical education hours was reduced in favor of increasing other subject matters. Nevertheless, it appears that 1-hour-per-week increase in physical education can only make a modest contribution to students' academic performance. Therefore, other complementary strategies, such as extracurricular or recreational sport, should be sought to increase the overall effects of exercise on the enhancement of students' performance.

An alternative solution to achieve an even greater increase in cognitive and school/work performance would be the combined use of the acute effects of exercise with the benefits of regular exercise. There are some indications suggesting that this combined intervention is more effective than their use alone. Hopkins et al. [136] found that a 4-week training program using aerobic exercise or an acute intervention using a single session of aerobic exercise (2 hours before the cognitive task) did not produce any improvement in a memory task performed at the end of the intervention. In contrast, the combination of both interventions (4-weeks training + acute exercise 2 hours before the memory task) led to a significant improvement in memory. Thus, these authors concluded that the effects of acute exercise were enhanced by the cognitive benefits of regular exercise, proposing that both forms of exercise intervention should be used together.

Other studies have applied strategies similar to that proposed Hopkins et al. [136], by implementing the performance of small doses of aerobic physical exercise (approx. 10 min) together with situations that require cognitive effort and repeated over several weeks, thus achieving an accumulation of the benefits of acute and regular exercise on cognition. One of the best examples of this strategy was carried out in the academic context [171]. Mahar et al. [171] showed an important positive impact when performing what they called “Energizers” (i.e., physical exercise activities in class that contributed to the teaching of the content reviewed in the theoretical subjects) several times a day and during a period of 12 weeks. The intervention through “Energizers” contributed significantly to the increase in the amount of physical activity that students did during the school hours, as well as benefiting students' attention to class assignments by 8%. Similarly, those students who usually showed a more deficient attention in class tasks improved their attention by 20%. Other studies have shown the benefits of several sessions of acute exercise practiced over several weeks on academic performance [172],[173] or work situations [174]–[176]. Thus, these types of strategies, that is, combining acute and regular exercise, could be one of the best ways to implement the potential benefits of physical exercise on cognition in academic and workplace situations.

## 5. Conclusions

This review examined the relationship between physical exercise and cognition. There are several cognitive benefits associated with the practice of both acute physical exercise and the practice of regular physical exercise on cognitive function. These positive exercise effects have been evidenced both in animal and in humans of adult and child age. Both, the acute benefits and those derived from regular exercise could be achieved from moderate to high intensity exercises of moderate duration. Even so, there are several moderating factors that can shape the exercise-cognition relationship, such as the level of physical condition of the person, the temporality of the exercise-cognitive task, or the type of cognitive task to be carried out, among others. Both, the acute and regular exercise benefits on cognition could be due to an improvement in synaptic activity, blood flow and brain irrigation, and an improvement in neuronal plasticity. Finally, several studies have implemented these exercise interventions in workplace and academic contexts, observing benefits on the individuals' work or academic performance, as well as on their health and well-being.
